# The oxidative stress paradigm in arbovirus infections: mechanisms and therapeutic insights

**DOI:** 10.1080/13510002.2026.2659994

**Published:** 2026-04-15

**Authors:** Chang Ke Yee, Rafidah Lani, Pouya Hassandarvish

**Affiliations:** a Department of Medical Microbiology, Faculty of Medicine, Universiti Malaya, Kuala Lumpur, Malaysia; b Tropical Infectious Diseases Research and Education Centre (TIDREC), Universiti Malaya, Kuala Lumpur, Malaysia

**Keywords:** Redox, oxidative stress, free radicals, Nrf2, mitochondria, arboviruses, therapeutics

## Abstract

**Background:**

Arbovirus infections impose a substantial global health burden, further complicated by their ability to induce oxidative stress through excessive generation of reactive oxygen species (ROS). This oxidative stress triggers a cascade that enhances viral replication and dysregulates immune responses, ultimately exacerbating disease pathology.

**Objective:**

In this review, we delineate the molecular pathways through which arbovirus-induced ROS activate NF-κB signalling, impair mitochondrial function, and alter the expression of key antioxidant enzymes (superoxide dismutase, catalase, and glutathione peroxidase), culminating in inflammatory tissue damage.

**Discussions:**

*In vitro* studies demonstrate that various alkaloids and polyphenols reduce viral load, while N-acetylcysteine has shown the ability to attenuate inflammation and reduce viral titres across both *in vitro* and *in vivo* models. Despite these advances, translation to clinical practice is constrained by limited compound bioavailability, variable pharmacokinetics, optimal timing windows, and a lack of standardized redox assays.

**Conclusion:**

We propose that targeted redox-modulating strategies, such as integrating genomic and metabolomic profiling, activating Nrf2 pathways, and incorporating advanced imaging techniques, warrant systematic evaluation using rigorous *in vivo* models and clinical trials. Defining optimal redox-directed interventions has the potential to catalyse the discovery of novel therapeutics that disrupt pro-viral oxidative pathways and improve outcomes in arboviral disease.

## 1. Introduction

Arboviruses constitute a diverse group of RNA viruses transmitted by hematophagous arthropods, causing significant morbidity and mortality in humans worldwide. The majority of outbreaks are driven by dengue virus (DENV), chikungunya virus (CHIKV), and Zika virus (ZIKV) [1]. Dengue alone accounts for an estimated 100–400 million infections annually, placing nearly half of the global population at risk of disease [2]. In 2023, CHIKV caused 620,000 reported cases and 213 fatalities across the Americas, Asia, Africa, and Europe [3], while ZIKV re-emerged in India in 2024 with 433 clinically suspected cases in three states [4]. Of the roughly 500 known arboviruses, approximately 134 are pathogenic to humans [5], with *Aedes aegypti* and *Aedes albopictus* serving as the principal vectors due to their peridomestic behaviour and extensive distribution in tropical, subtropical, and temperate regions [6]. *Ae. aegypti* typically exhibits higher DENV transmission efficiency, while *Ae. albopictus* possesses a stronger salivary gland barrier that limits the amount of virus in saliva [7]. *Ae. aegypti* is highly competent for all ZIKV lineages, but *Ae. albopictus* shows lower and more heterogeneous transmission rates, particularly for Asian/American lineages [8]. Although historically *Ae. aegypti* was the primary vector for CHIKV, a single mutation (E1-A226V) in the virus dramatically increased its competence in *Ae. albopictus*, allowing it to drive massive outbreaks in 2005 Réunion Island outbreak [9]. Clinical manifestations span from self-limited febrile illness to severe dengue haemorrhagic fever, debilitating chikungunya arthritis, and Zika-associated congenital anomalies. Yet, no specific antiviral therapies are approved, and vector control measures remain insufficient to interrupt transmission. This unmet need highlights the importance of dissecting virus-host interactions, particularly oxidative stress pathways, to uncover host-directed strategies for mitigating arboviral disease.

**Figure 1.**
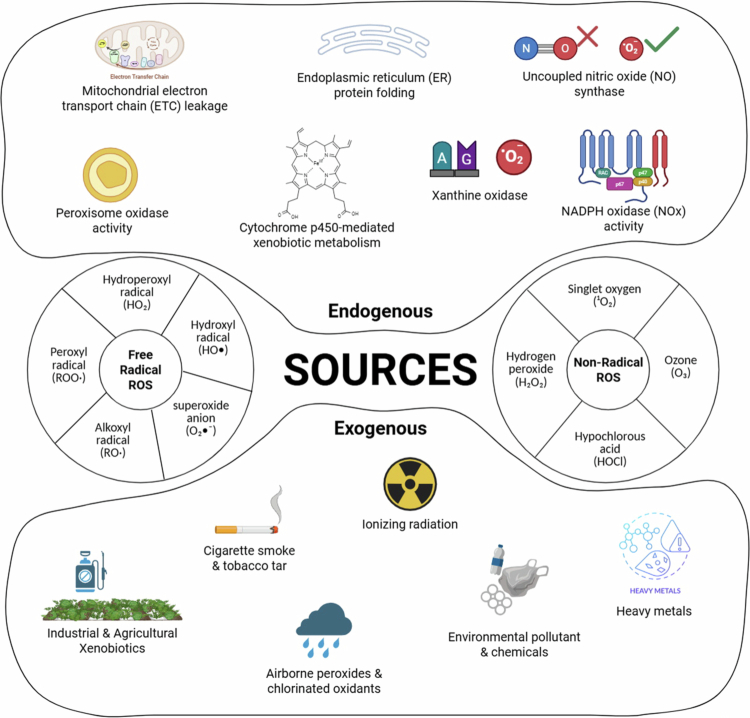
Species of free radical and non-free radical ROS, and their sources from the environment and human biological systems. This image was created using BioRender.com.

A state of oxidative stress is reached when the accumulation of reactive oxygen species (ROS) exceeds the functional threshold of enzymatic and non-enzymatic defences, leading to a sustained shift in the cellular redox environment [10]. While these reactive molecules are fundamental to physiology, their overproduction is driven by specific internal mechanisms, such as electron leakage during mitochondrial transport and the oxidative bursts of activated leukocytes [11]. External factors, ranging from environmental pollutants to xenobiotic exposure, serve to exacerbate this imbalance, pushing the cell toward a state of oxidative overload [12]. ROS include free radicals and non-radical species (Figure 1), with non-radical ROS being less reactive than free radicals, yet still are highly reactive and able to participate in redox reactions [13]. Low levels of ROS are essential for normal cell function. For example, exercise-induced ROS helps regulate muscle tension by modulating contraction and relaxation [14]. Under basal conditions, ROS act as signals for cell division, differentiation, and immune activation [15]. Nitric oxide (NO) is a key radical that controls blood flow, clotting, immune defence, and neuronal signalling [16]. To harness the beneficial roles of ROS while preventing cellular damage, cells rely on redox reactions and antioxidant systems to maintain redox homeostasis.

However, when ROS production exceeds the buffering capacity of antioxidant defences, their high reactivity inflicts irreversible damage on cellular macromolecules; oxidizing lipids, denaturing proteins, fragmenting carbohydrates, and inducing DNA lesions, which compromise membrane integrity, enzyme function, and genomic stability, ultimately triggering cell dysfunction and death [17–19]. Chronic oxidative stress underpins a spectrum of pathological processes, from accelerated ageing and sustained inflammation to degenerative disorders [20]. In cancer, persistent ROS overproduction drives genomic instability, promotes oncogenic signalling, and facilitates tumour initiation, progression, and metastasis [21,22]. In cardiovascular disease, excessive ROS impairs endothelial NO bioavailability, increases vascular resistance, and exacerbates hypertension and atherogenesis [23]. Moreover, in neurodegenerative diseases such as Alzheimer's, Parkinson's, and Huntington's, elevated ROS levels contribute to synaptic failure, protein misfolding, and neuronal loss, thereby fuelling cognitive decline and motor dysfunction [24].

Recent advancements in molecular pathology have highlighted the role of redox-sensitive mitochondrial remodelling as a primary driver of emerging paradigms of cell death. Beyond traditional apoptosis, She et al. (2025) recently identified a mechanistic axis wherein Arginase 1 (Arg1) interacts with Mic10 to drive mitochondrial cristae disorder, a process further exacerbated by hypoxia-induced VDAC1 lactylation and the subsequent opening of the mitochondrial permeability transition pore (MPTP) [25]. This structural failure facilitates the translocation of mitochondrial DNA (mtDNA) into the cytosol, where it activates the cGAS-STING pathway to trigger PANoptosis, a highly inflammatory, integrated cell death process encompassing features of pyroptosis, apoptosis, and necroptosis [25].

In the context of arboviral pathogenesis, this mitochondrial-redox framework provides a robust explanation for the synchronized tissue damage observed during infection [26]. Upon invasion, phagocytic cells produce an oxidative burst to eliminate virions, yet many viruses exploit elevated ROS to enhance replication and evade immune detection [27]. Excess ROS activates redox-sensitive transcription factors, including NF-κB and activator protein-1, leading to heightened pro-inflammatory cytokine release and sustained tissue damage [28]. Oxidative modifications of lipids, proteins, and nucleic acids compromise cellular membranes, impair vascular integrity, and trigger apoptosis [29]. DENV infection elicits NADPH oxidase-driven and mitochondrial ROS overproduction, activating NF-κB and promoting TNF-α/IL-6 release that exacerbates endothelial dysfunction and plasma leakage [30]. CHIKV triggers mitochondrial and ER stress-associated ROS in synovial fibroblasts, leading to inflammasome activation, caspase-1-dependent cytokine release, and chronic joint damage [31]. ZIKV exploits host redox pathways in neural progenitors, where excessive mitochondrial ROS and reduced catalase/glutathione peroxidase activities drive lipid peroxidation, DNA breaks, and apoptosis, underpinning neurodevelopmental defects [32]. In this review, we delineate how arbovirus-induced ROS activate NF-κB, impair mitochondrial function, and dysregulate key antioxidant enzymes to drive inflammatory tissue damage, and we assess the efficacy of pre-clinical antioxidant interventions. Elucidating these redox-dependent mechanisms is essential for developing adjunctive antioxidant strategies to restore homeostasis and limit virus-induced injury. This review distinguishes itself from the prior reviews by integrating the interplay between NF-κB activation and mitochondrial dysfunction, rather than treating them as separate events. The review further differentiates itself through a focused, cross-family, paradigm-based approach to antioxidant intervention as adjunctive, rather than merely general, therapies.

## 2. Search strategy, study selection, and data extraction and synthesis

**Figure 2.**
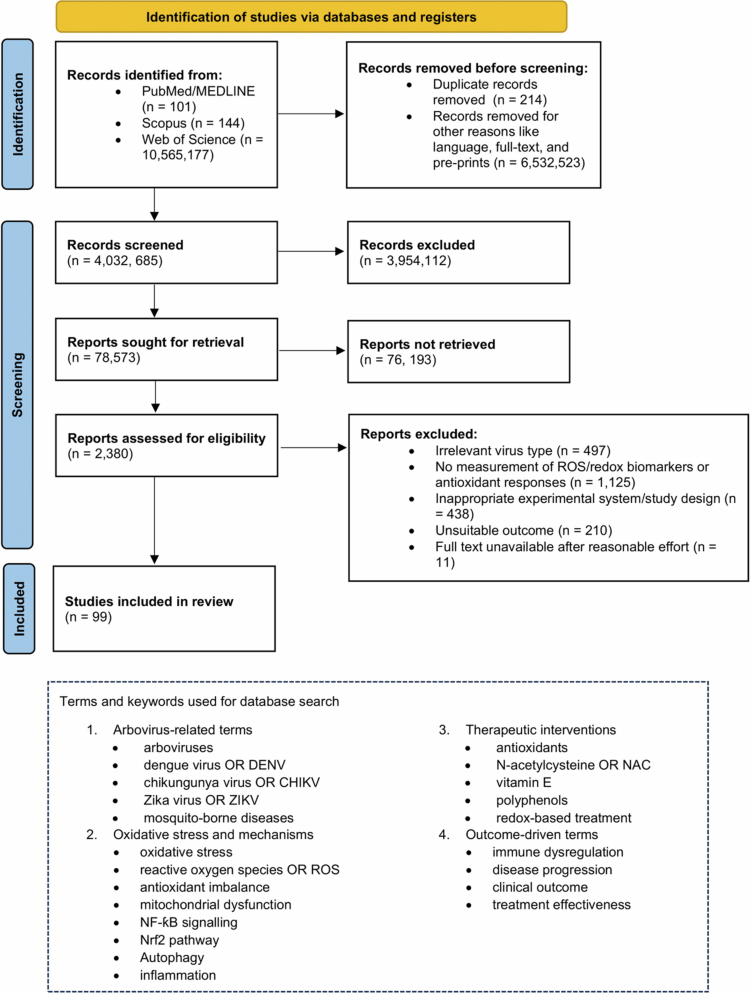
PRISMA 2020 flow diagram of study selection for the systematic search strategy on oxidative stress in arbovirus infections. After applying language and publication-type filters, removing duplicates, and screening titles/abstracts, full-text assessment was performed. Ultimately, 99 studies met the inclusion criteria and were included in the qualitative synthesis. Reasons for exclusion are detailed in the diagram.

Systematic literature searches were conducted in accordance with the Preferred Reporting Items for Systematic Reviews and Meta-Analyses (PRISMA) guidelines (Figure 2). Three electronic databases, PubMed/MEDLINE, Scopus, and Web of Science, were queried from their inception through 30 June 2025. Search terms combined Medical Subject Headings (MeSH) and free-text keywords were used in the core search string (PubMed), and Boolean operators (AND/OR) and database-specific field tags were adapted for Scopus and Web of Science. All identified records were imported into EndNote X9 for deduplication. Two reviewers (C.K.Y., R.L.) independently screened titles and abstracts against predefined criteria. Full-text articles were retrieved for potentially eligible studies and assessed in duplicate. Discrepancies were resolved by consensus or arbitration by a third reviewer (P.H.). Extracted data based on virus and experimental system, ROS assays, and key redox biomarkers, molecular mechanism readouts, therapeutic agent, and reported outcomes were tabulated and synthesized qualitatively to map oxidative mechanisms and quantitatively, where homogeneity permitted.

## 3. Mechanisms of oxidative stress in DENV, CHIKV, and ZIKV infections

### 3.1. Endothelial barrier dysfunction during DENV infection

DENV initially targets vascular endothelial cells (ECs), the principal barrier to systemic spread. Upon attachment and entry, DENV hijacks host ribosomes to translate its positive‐sense RNA genome and assemble progeny virions, a process essential for viral amplification and dissemination beyond the vasculature [33]. In ECs, DENV infection elicits a rapid surge in ROS derived from both NADPH oxidase (NOX) complexes (principally NOX2 and NOX4) and dysfunctional mitochondria [30,34]. NOX-derived ROS augment viral replication and disrupt intercellular junctions, undermining barrier integrity [30]. Evidence confirms that DENV triggers a synchronized ROS surge through the activation of NOX2 and NOX4 complexes and the disruption of mitochondrial membrane potential, with the latter often mediated by viral non-structural proteins [35]. This oxidative environment serves a dual purpose for the virus, where it facilitates viral replication by inducing autophagy and lipid remodelling, while simultaneously triggering Src kinase-mediated internalization of VE-cadherin [36]. These combined mechanisms lead to a measurable collapse of the endothelial barrier, providing a clear mechanistic basis for the vascular leakage and inflammatory signalling (such as NLRP3 activation) that define severe disease [37].

The DENV NS2B3 protease complex targets nuclear factor erythroid 2-related factor 2 (Nrf2) for lysosomal degradation, suppressing antioxidant gene networks, including superoxide dismutase, catalase, and glutathione peroxidase, and driving progressive ROS accumulation that correlates with increased viral replication and inflammatory gene expression [38,39]. Accumulated ROS serve as second messengers that activate IRF3-STAT1 and NF-κB signalling pathways, driving type I interferon (IFN) responses and p53-mediated apoptosis as innate antiviral defences [40]. Simultaneously, mitochondrial perturbations evidenced by diminished maximal respiratory capacity and collapse of membrane potential elevate mitochondrial ROS (mtROS), further promoting EC death and vascular leakage [41]. Heightened ROS also triggers secretion of pro-inflammatory cytokines (CCL5, TNF-α, IL-6, IL-8), fuelling a cytokine storm and amplifying microvascular inflammation [42]. At the transcriptional level, DENV strategically disrupts the Nrf2-antioxidant response. Transcriptomic analyses of DENV-infected ECs confirm downregulation of Nrf2-responsive genes alongside upregulation of oxidative stress markers and pro-inflammatory mediators [40]. Oxidative modifications of high-mobility group box 1 (HMGB1) under sustained ROS conditions impair its regulatory functions and destabilise tight junction proteins (VE-cadherin, ZO-1), thereby exacerbating plasma leakage and predisposing to haemorrhage and hypotension characteristic of severe dengue [43]. Under sustained oxidative stress, HMGB1 undergoes a critical conformational change into its disulphide form (Cys23-Cys45), transforming it from a nuclear DNA-binding protein into a potent extracellular alarmin [44]. This oxidized HMGB1 binds to TLR4 and RAGE receptors on the endothelial surface, triggering a signalling cascade that activates Src family kinases [44]. The mechanistic consequence is rapid phosphorylation and internalization of VE-cadherin and the reorganization of ZO-1, which are the master regulators of paracellular permeability [45]. While VE-cadherin maintains the structural zipper of the adherens junction, ZO-1 acts as a crucial scaffold protein that anchors the tight junction complex to the actin cytoskeleton. Their simultaneous disruption leads to the physical collapse of intercellular barriers. Clinically, this transition from a localized immune response to systemic junctional failure provides a primary mechanistic explanation for the sudden plasma leakage, hypotension, and hypovolemic shock observed in the critical phase of severe dengue [46].

### 3.2. CHIKV-induced polyarthralgia and chronic arthritis

CHIKV infection precipitates a profound oxidative insult in joint tissues that underlies the characteristic polyarthralgia and chronic arthritis in affected patients [47]. Early after infection, CHIKV disrupts mitochondrial electron transport chain complexes I and III in synovial fibroblasts, causing electron leakage, collapse of mitochondrial membrane potential, and excessive mtROS production [48]. This surge in mtROS triggers cytochrome c release and intrinsic apoptosis of chondrocytes and synoviocytes, thereby compromising cartilage and synovial integrity [49]. CHIKV non-structural proteins co-opt NADPH oxidase complexes NOX2 and NOX4, amplifying superoxide anion generation at the plasma membrane [50]. NOX-derived ROS oxidize cell-surface thiols and weaken endothelial tight junctions in periarticular microvessels, promoting vascular leakage, oedema, and increased viral dissemination within joint spaces [51]. The combined mitochondrial and NOX-driven oxidative burden overwhelms the depleted antioxidant defences, owing in part to CHIKV-mediated downregulation of the Nrf2 pathway and reduced expression of superoxide dismutase, catalase, and glutathione peroxidase, resulting in accumulation of lipid peroxidation products such as malondialdehyde (MDA) and protein carbonyls in cartilage and bone matrices [52]. These heightened ROS levels function as second messengers that activate redox-sensitive signalling cascades, including NF-κB and p38 MAPK, within macrophages and fibroblast-like synoviocytes (FLSs) [53].

Downstream of these pathways, there is robust upregulation of proinflammatory cytokines (TNF-α, IL-6, IL-8) and matrix metalloproteinases (MMP-1, MMP-3), which perpetuate synovial inflammation and degrade extracellular matrix components [54]. A clear mechanistic link between MMPs and the irreversible structural failure of articular cartilage was established in recent studies. The process is led by MMP-13, which is uniquely capable of cleaving the Type II collagen triple helix, thereby destroying the cartilage's tensile framework [55]. This destruction is amplified by MMP-3, which not only degrades proteoglycans like aggrecan, leading to a loss of tissue hydration and compressive resistance, but also serves as a critical activator for other latent pro-MMPs [56]. Computational models highlight that the enzymatic cascade is often triggered by mechanical shear and oxidative stress, which drive chondrocytes to shift from a homeostatic state of catabolic phenotype [57]. The resulting loss of the superficial collagen layer and the depletion of the aggrecan matrix result in the joint space narrowing and subchondral bone changes characteristic of advanced osteoarthritis.

Oxidative stress also impairs innate immunity by inducing Siglec-9 overexpression on monocytes and neutrophils [58], thereby attenuating phagocytosis and antiviral cytokine production and facilitating CHIKV persistence in joint tissues. CHIKV-driven ROS alter the epigenetic landscape of bone marrow-derived mesenchymal stem cells (BMSCs), modifying DNA methylation and histone acetylation patterns that bias differentiation toward a pathogenic FLS phenotype [59]. These aberrant FLSs secrete chemokines such as CCL2 and CXCL10, further recruiting inflammatory cells and driving structural damage to bone and cartilage, a process that underpins the transition from acute arthralgia to debilitating chronic arthritis [60].

### 3.3. Oxidative stress in ZIKV-associated microcephaly

ZIKV infection profoundly disrupts mitochondrial integrity in neural cells, triggering excessive mtROS production that underlies lipid peroxidation, protein oxidation, and DNA damage [61]. In human iPSC-derived astrocytes and neural progenitor cells, ZIKV promotes Drp1-mediated mitochondrial fragmentation while downregulating mitofusin-2, leading to collapse of mitochondrial membrane potential and impaired ATP synthesis [62]. This dysfunction drives a surge in mtROS, which correlates with elevated MDA and protein carbonyl levels in both cultured cells and ZIKV-infected mouse brains, as well as increased γH2AX foci marking DNA double-strand breaks [63]. Pharmacological inhibition of mitochondrial fission by Mdivi-1 restores mitochondrial network integrity, reduces mtROS accumulation, and attenuates ZIKV-induced neuronal apoptosis, demonstrating a causal role for mitochondrial dynamics in ZIKV neuropathogenesis [64]. ZIKV infection also perturbs the host antioxidant defence system through complex modulation of the Nrf2 pathway.

Early during infection, Nrf2 is transiently activated as a compensatory response; however, sustained ZIKV replication ultimately suppresses Nrf2 transcriptional activity, resulting in diminished expression of key antioxidants, including superoxide dismutase (SOD), catalase, and glutathione peroxidase [65]. A biphasic Nrf2 response is a critical molecular pivot governing ZIKV pathogenesis. Initially, the host initiates a transient, compensatory Nrf2 surge to counteract early oxidative stress. However, as ZIKV replication intensifies, the virus actively subverts this defence, likely through non-structural protein interference, leading to the sustained suppression of Nrf2 transcriptional activity [65]. The resulting collapse of the antioxidant network, characterized by the profound depletion of SOD, catalase, and glutathione peroxidase, transitions the cellular environment into a state of uncontrolled oxidative injury [66]. Translationally, this redox collapse serves as a primary driver of the apoptosis in neural progenitor cells linked to microcephaly [67], suggesting that the clinical efficacy of Nrf2-targeted therapies is strictly dependent on the timing of intervention during the infection cycle.

Inhibition of Nrf2 or depletion of glutathione enhances viral replication, whereas pharmacological Nrf2 inducers and glutathione precursors restore redox balance and impair ZIKV propagation in neural and hepatic models [65]. Intriguingly, in placental trophoblast-like cells, ZIKV-host interactions can transiently boost Nrf2 signalling and antioxidant enzyme levels, suggesting cell-type-specific differences in redox engagement that may influence vertical transmission risk [68]. Endoplasmic reticulum (ER) stress and activation of the unfolded protein response (UPR) further amplify oxidative injury during ZIKV infection. Accumulation of misfolded viral polyproteins in the ER lumen activates PERK, IRE1α, and ATF6 branches of the UPR, leading to upregulation of CHOP and phosphorylation of eIF2α [69]. The resulting translational arrest and sustained UPR signalling converge with mtROS and genotoxic stress to stabilize and activate p53, promoting intrinsic apoptosis of neural progenitors and astrocytes [70].

The translational relevance of murine models to ZIKV-induced microcephaly has successfully replicated the placental-foetal axis of human infection. Considering ZIKV cannot naturally bypass the murine interferon response, researchers primarily utilize immunodeficient models, such as the interferon α/β receptor knockout (*Ifnar*1^−/−^) or the interferon α/β and γ receptor knockout (AG129) mice [71,72]. These models have definitely shown that ZIKV crosses the placenta, infects radial glial cells (neural progenitor cells), and induces severe cortical thinning. Recent research has further refined this by using wild-type mice with transient IFN-blockade (via anti-Ifnar1 antibodies) or in utero electroporation to study the virus's impact on the developing neuroepithelium [73]. These models confirm that the primary driver of microcephaly is a virus-induced redox collapse, specifically the transition from transient Nrf2 protection to sustained mitochondrial-dependent apoptosis. By mirroring the human clinical progression of intrauterine growth restriction and neurodevelopmental arrest, these specific murine systems have become the gold standard for testing therapeutics intended to stabilize mitochondrial integrity and prevent the massive loss of neural progenitor cells that results in microcephaly.

ZIKV-infected murine models exhibit increased p53 accumulation, elevated markers of ER stress, and widespread neuronal cell death, linking ER-mitochondrial crosstalk to microcephaly and neurodevelopmental defects [74]. Beyond direct oxidative damage, ZIKV-induced ROS serve as second messengers that drive neuroinflammation. Rather than causing immediate structural damage, mtROS and NOX-2-derived superoxide selectively activate MAPK and NF-κB pathways within microglia and astrocytes, orchestrating a potent pro-inflammatory secretome [75]. This cascade specifically upregulates chemotactic ligands like CCL2 and CXCL10, which are instrumental in compromising the blood-brain barrier and recruiting peripheral immune cells into the CNS. *In vivo* evidence highlights that the resulting systemic depletion of superoxide dismutase and catalase activities creates a state of redox exhaustion, which sustains a chronic neuroinflammatory loop [76]. This shift from localized oxidative stress to a self-perpetuating inflammatory environment is a primary driver of the impaired neurogenesis and long-term neurological sequelae observed in neonatal ZIKV models.

## 4. Antioxidant and redox-based interventions

Prophylactic and therapeutic redox strategies differ based on their temporal relationship to the redox tipping point. Prophylactic strategies focus on priming the host's endogenous defences, primarily through the induction of the Nrf2-ARE pathway, to enhance the cellular oxidative buffer before an insult occurs [77]. In contrast, therapeutic strategies are reactive, utilizing direct ROS scavengers or mitochondria-targeted antioxidants to neutralize existing superoxide surges and arrest the feed-forward loops of inflammation and paracellular gap formation once damage is underway [78]. The clinical consensus suggests that while prophylaxis targets the underlying homeostatic capacity, the efficacy of therapeutic intervention is strictly time-dependent. Late-stage administration often fails to reverse the downstream inflammatory cascades, such as PANoptosis and irreversible junctional collapse, that have already set in motion [79].

**Figure 3.**
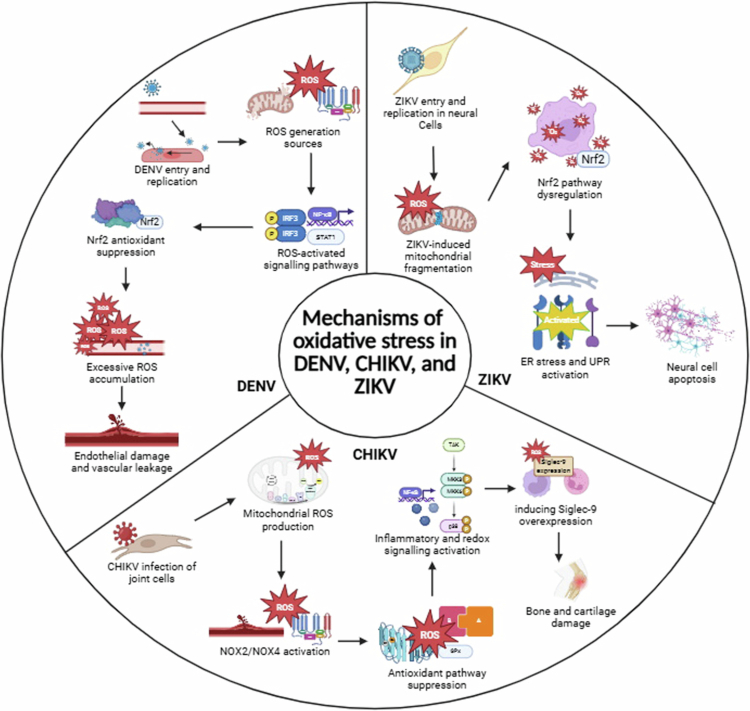
Mechanisms of oxidative stress in DENV, CHIKV, and ZIKV infection, known to exacerbate the pathogenesis of the diseases. This image was created using BioRender.com.

Several promising antioxidant- and redox-modulating strategies (Table 1) have emerged in the past few years to counteract the virus-driven oxidative damage that exacerbates DENV, CHIKV, and ZIKV pathogenesis (Figure 3). By targeting the imbalance between pro-oxidants and antioxidants, these interventions can attenuate cytokine storms, preserve cellular integrity, and improve clinical outcomes.

**Table 1.** Selected studies evaluating promising antioxidant-antiviral and redox modulating strategies relevant to DENV, CHIKV, and ZIKV using different platforms.

No.	Virus or subject	Compound or drug (platform)	Strength(s)	Limitation(s)	Ref.
1	DENV-2	Glutathione (HepG2-grafted SCID mice)	Humanized liver environment with the use of human hepatoma (HepG2) cells engrafted into SCID mice, which effectively bypasses the natural resistance of murine hepatocytes to DENV infection	Complete lack of an adaptive immune system. The study cannot predict whether antioxidant intervention would interfere with or be overwhelmed by the adaptive immune system in a human host	[80]
2	DENV-1, 2, 3, and 4	N-acetyl cysteine (HepG2 cells and AG129 mice)	Robust mechanistic and translational foundation for repurposing an FDA-approved drug. NAC does not merely act as a passive ROS scavenger; it actively restricts DENV replication across multiple serotypes	ROS are essential secondary messengers required to trigger the IFN-mediated innate immune response; the AG129 model cannot evaluate whether NAC might inadvertently blunt the host's endogenous viral restriction signals	[81]
3	DENV-1, 2, 3, and 4	Melatonin (Huh7 cells)	This research demonstrates that melatonin acts as a signalling modulator that promotes the phosphorylation of STAT1 and the subsequent expression of ISGs	No corresponding validation in an animal model and the study does not address the pharmacokinetic challenges of achieving and maintaining the specific micromolar concentrations of melatonin required to activate SIRT1 within target cells	[82]
4	Children with dengue fever	Co-administration of Vitamin C and E (double-blind, randomized, placebo-controlled trial)	High-quality clinical evidence for a demographic that is disproportionately affected by severe dengue. It provides a low-cost, scalable therapeutic strategy to manage thrombocytopenia in resource-limited settings where standard-of-care options for managing platelet counts	The study may lack granular analysis of critical confounding variables, such as the exact timing of the critical phase relative to supplementation or the potential for high-dose antioxidants to interfere with the ROS-dependent innate immune signalling	[83]
5	CHIKV	Small-molecule inhibitor MCC950 (Peripheral blood mononuclear cells isolated from CHIKV-infected patients and the C57BL/6 mice)	High-resolution mapping of the NLRP3-caspase-1-IL-1β as the definitive orchestrator of alphavirus-induced musculoskeletal pathology. Targeted suppression of the specific inflammasome complex effectively attenuates joint swelling, inflammatory cell infiltration, and subsequent bone resorption in CHIKV-infected mice	It does not fully address whether the inflammasome-mediated production of IL-1β and the induction of pyroptosis are necessary components of the host's early defence to restrict viral replication and dissemination	[84]
6	CHIKV	Lycorine (Vero and HeLa cells)	Lycorine inhibits the alphaviruses at remarkably low, nanomolar concentrations (low EC_50_ values) while maintaining a high Selectivity Index (SI). This positions the alkaloid as a highly potent lead candidate for the development of pan-alphaviral therapeutics	The study does not provide animal model validation to determine if these therapeutic concentrations can be safely achieved in systemic circulation	[85]
7	CHIKV	Berberine, abamectin, and ivermectin (BHK-21, CHIKV replicon containing cell line (BHK–CHIKV), and Huh-7.5 cells)	High-throughput screening provides a high-confidence shortcut for drug repurposing, identifying existing chemical scaffolds that can be immediately studied for their broad-spectrum activity across the *Alphavirus* genus	The high micromolar concentrations (IC_50_ values) required to achieve effective viral inhibition *in vitro* may not be safely attainable in human patients. Accompanying *in vivo* data or pharmacometric modelling are necessary	[86]
8	ZIKV	Sofosbuvir and type-I interferons (α and β) in synergy (Huh7 cells)	Checkerboard titration method and MacSynergy II analysis demonstrated that combining these agents achieves significantly greater viral inhibition than the sum of their individual effects. Synergy allows for the use of lower clinical doses, potentially reaching therapeutic efficacy against ZIKV while minimizing the severe systemic side effects and complications often associated with high-dose interferon monotherapy	Cells used are not the primary physiological targets of ZIKV infection. It may not translate to the neural progenitor cells (NPCs) or placental tissues where ZIKV-induced damage is most severe. The critical pharmacokinetic hurdle of blood-brain barrier (BBB) penetrance for Sofosbuvir needs to be addressed	[87]
9	DENV-2	Eltrombopag (Molecular docking and dynamics simulation, BHK-21 and Huh7 cells, AG129 mice, and Sprague-Dawley rats)	Identification of Eltrombopag as a non-competitive, allosteric inhibitor of the NS2B-NS3 protease, a critical enzyme for flavivirus polyprotein processing. It possesses unique ‘dual-action’ potential: it could theoretically arrest viral replication while simultaneously addressing the thrombocytopenia that defines Dengue Haemorrhagic Fever (DHF)	Eltrombopag is a known thrombopoietin receptor agonist (TPO-RA) with potential liver injury; since DENV is inherently hepatotropic and causes significant oxidative liver damage	[88]
10	CHIKV	Direct-Acting Antivirals (DAAs) (Molecular docking and dynamics simulation, and Vero cells)	Integrating structural bioinformatics with *in vitro* assays significantly narrows the chemical space for subsequent experimental validation and reduces the time and cost associated with traditional drug discovery	High theoretical binding affinity does not always correlate with a functional reduction in viral titres or a favourable safety profile in human tissues	[89]
11	ZIKV	Montelukast (BHK-21, Vero, RD, and U-251 MG cells, and AG129 mice)	Montelukast disrupts the integrity of the virion and irreversibly inhibiting viral infectivity	The virucidal concentrations used *in vitro* and *in vivo* may be difficult to reach in human patients, especially in the brain or placenta, without administering doses that could lead to systemic toxicity or neuropsychiatric side effects	[90]

In DENV infection, pre-clinical studies in human endothelial and immune cells have shown that N-acetylcysteine (NAC) supplementation replenishes intracellular glutathione, reduces lipid peroxidation, and lowers levels of proinflammatory cytokines such as TNF-α and IL-6 [30]. In SCID mice with a humanized liver environment, administration of exogenous glutathione significantly reduced MDA levels and restored the activity of antioxidant enzymes (CAT and T-SOD), resulting in much milder liver injury compared to untreated DENV-infected mice [80]. Recent advances include PEGylated NAC nanocarriers that sustain glutathione delivery and markedly improve endothelial barrier integrity in DENV-infected mice [81], as well as high-dose melatonin regimens [82] that inhibit NADPH oxidase, suppress ROS bursts, and reduce plasma leakage in pre-clinical studies. Co-administration of vitamin C and lipid-soluble vitamin E analogues further attenuates oxidative damage by scavenging superoxide radicals and inhibiting NADPH oxidase activity [91], although clinical translation remains under investigation.

While vitamin C and E are broad-spectrum antioxidants, their specific relevance to DENV centres on their ability to disrupt the oxidative-inflammatory loop that drives vascular leakage. Vitamin C acts within the aqueous phase to neutralize NOX-derived superoxide and protect the endothelial glycocalyx, while vitamin E analogues partition into lipid membranes to arrest the lipid peroxidation that destabilizes VE-cadherin and ZO-1 complexes [83]. The mechanistic strength of this pairing lies in the redox recycling capacity of vitamin C to regenerate vitamin E, maintaining a continuous barrier against the sustained oxidative burst of the DENV critical phase [92]. However, clinical translation remains a challenge. While these interventions successfully attenuate biomarkers of oxidative damage, their ability to reverse established plasma leakage once the inflammatory tipping point has been reached is currently the focus of ongoing trial optimization [93].

CHIKV-induced arthralgia and myositis are strongly associated with mitochondrial dysregulation and ROS-mediated activation of inflammasomes in synovial and muscle tissues [94]. *In vitro* exposure of CHIKV-infected fibroblasts and epithelial cells to NAC [95] and α-tocopherol [96] mitigates mitochondrial membrane depolarization, lowers mitochondrial ROS production, and suppresses caspase-1 activation. In mouse models of CHIKV arthritis, oral NAC reduces joint swelling, decreases expression of NLRP3 inflammasome components, and preserves cartilage integrity [84,97]. Emerging approaches that activate the Nrf2 pathway, such as sulforaphane (an electrophilic Nrf2 activator) administration, upregulate a suite of phase II detoxifying enzymes [98], offering a multi-targeted means to restore redox homeostasis during CHIKV infection. Daily sulforaphane dosing upregulates HO-1 [99] and NQO1 [100], lowers synovial ROS, and preserves cartilage architecture in the osteoarthritis *ex vivo* model [100] and animal model [101].

The relevance of flavonoid glycosides to antioxidant-antiviral synergy lies in their structural complexity, which allows them to modulate both the viral life cycle and the host's intracellular redox environment. Unlike simple aglycones, the sugar moieties in glycosides often enhance solubility and bioavailability, facilitating more efficient interaction with cellular targets during infection [102]. This synergy is especially relevant for CHIKV, where the restoration of GSH levels and the inhibition of lipid peroxidation are vital to preventing the transition from acute infection to chronic synovial inflammation [47]. Plant-derived alkaloids lycorine [85] and berberine [86] display dual antiviral and antioxidative actions against CHIKV, restoring glutathione pools and inhibiting lipid peroxidation *in vitro*. By complementing the action of alkaloids like lycorine and berberine, flavonoid glycosides offer a broad-spectrum approach to mitigating both viral load and the oxidative bystander damage that drives clinical joint pathology.

ZIKV neuropathogenesis is similarly attenuated by redox-based interventions. In ZIKV-infected neural cells, dysregulated redox signalling underlies apoptosis, DNA damage, and developmental defects. Treatment with NAC reversed these oxidative insults and improved cell survival in neural progenitor cultures [103]. Pharmacological agonism of PPAR-γ and upregulation of PGC-1α potentially could be repurposed to tackle ZIKV-induced oxidative stress in neural cell systems [104]. Activation of PPAR-γ not only corrected dysregulated lipid metabolism but also enhanced expression of antioxidant genes [105], reducing ROS-driven apoptosis in ZIKV-infected neural progenitors. PPAR-γ agonists such as rosiglitazone enhance antioxidant gene expression and mitochondrial bioenergetics [106], suggesting potential to counteract ZIKV-driven p53 activation and neuronal death. Boosting PGC-1α-mediated mitochondrial biogenesis could restore mitochondrial membrane potential and lower ROS accumulation in human astrocytes [107] exposed to ZIKV, thereby preserving genomic integrity and preventing premature cell cycle arrest. Mitochondria-targeted peptide SS-31 [108] directly scavenges mtROS, restores membrane potential, and might be able to suppress viral replication in neural cells. Moreover, combinatorial regimens pairing sofosbuvir and type‐I interferons exhibit synergistic reductions in viral titres and oxidative damage *in vitro* [87], setting the stage for future clinical evaluation.

Collectively, these findings support a paradigm in which antioxidant and redox-modulating therapies can complement antiviral strategies. By combining direct-acting antivirals with targeted enhancement of endogenous antioxidant responses, through NAC, Nrf2 activators, PPAR agonists, or PGC-1α modulators, it may be possible to mitigate both viral replication and collateral oxidative damage, ultimately improving outcomes in DENV, CHIKV, and ZIKV infections.

## 5. Translational potentials and limitations of redox-based therapeutics

Redox-based therapies offer a compelling adjunct to direct‐acting antivirals by targeting the host oxidative burst that underlies vascular leakage in DENV, chronic arthritis in CHIKV, and neuroinflammation in ZIKV. In dengue fever, thiol donors such as NAC have demonstrated robust pre-clinical efficacy: nanoparticle-encapsulated NAC achieves sustained intracellular glutathione replenishment, reverses endothelial hyperpermeability, and reduces haemorrhagic signs in murine models [81]. Mitochondria-targeted antioxidants such as MitoQ have shown promise in attenuating flow leak and apoptosis-driven endothelial syndrome in murine models, normalizing barrier integrity [109]. Thiol donors and mitochondrial antioxidants have shown efficacy in pre-clinical dengue models, but their narrow therapeutic window challenges clinical translation.

The clinical translation of antioxidant-antiviral co-therapies is constrained by a narrow therapeutic role and the dualistic physiological roles of ROS. A primary obstacle is the temporal discordance in ROS function. While early-phase oxidative bursts are requisite for innate immune signalling and type I interferon induction, sustained late-phase production drives systemic oxidative stress and pathological tissue damage [110], such as vascular leakage and chronic synovial inflammation. This complexity is compounded by the biphasic, U-shaped dose-response curves of many redox modulators [111], which risk pro-oxidant activity or the inhibition of host-protective apoptosis at supratherapeutic concentrations. The absence of organ-specific, real-time redox biomarkers complicates precise dosing, a challenge exacerbated by viral subversion strategies that may paradoxically benefit from exogenous antioxidant support [112]. The significant pharmacokinetic discrepancy between *in vitro* potency and *in vivo* bioavailability persists as a major barrier, as achieving therapeutic concentrations in target microenvironments often requires dosages that approach systemic toxicity thresholds.

Excessive antioxidant dosing may blunt ROS-dependent antiviral defences, prolonging viraemia and heightening secondary infection risk. In the context of arboviral infections, the therapeutic administration of high-dose antioxidants risks compromising the host's innate antiviral response by neutralizing obligate ROS signalling. While pathological oxidative stress facilitates tissue injury, a physiological oxidative burst serves as a critical secondary messenger for the activation of pattern recognition receptor (PRR) signalling pathways, specifically the phosphorylation of IRF3, IRF7, and STAT1 required for type I interferon induction [113]. Pharmacological suppression of these redox switches has been shown to dampen the expression of interferon-stimulated genes (ISGs), thereby facilitating increased viral replication and higher titres [114].

Furthermore, the attenuation of ROS-dependent, mitochondrial-mediated apoptosis by exogenous antioxidants may inadvertently extend the viability of infected cells [115], providing a safe harbour for sustained viral assembly and dissemination. This effect is compounded by the blunting of paracrine redox signals required for maturation of uninfected bystander cells marked by CD83 upregulation, which diminishes tissue-wide resistance to viral spread [116]. Given that DENV and ZIKV have evolved specific mechanisms to actively subvert host Nrf2-mediated proteostasis [117], excessive antioxidant intervention may paradoxically harmonize with viral survival strategies, facilitating positive-strand RNA replication by disarming the host's endogenous redox-dependent restriction factors. Harnessing liposomal formulations of *α*-tocopherol and coenzyme Q10 can further enhance tissue distribution [118], suggesting a pathway to combine these compounds with existing antivirals to blunt immunopathology while preserving host defence.

High-dose melatonin, with its dual antioxidant and anti-inflammatory actions, has shown promise in phase I studies to dampen NADPH oxidase–driven ROS surges and improve microvascular function in sepsis conditions [119]. These translational successes hinge on optimized delivery systems, PEGylation for half-life extension [120], liposomal formulations for targeted endothelial uptake [121], and mitochondria-specific dyes to monitor redox biomarkers for dose titration [122]. Interpatient variability in oxidative baseline, driven by genetic polymorphisms [123], nutritional status [124], and co-morbidities [125], complicates dose optimization and biomarker-guided titration. Moreover, validated endothelial endpoints (for example, capillary leakage indices) are lacking in phase II trials [126], leaving clinicians without standardized criteria for initiating or escalating redox therapy.

Nrf2 activators such as sulforaphane and bardoxolone methyl have moved rapidly from cell culture to *in vivo* validation, where they restore phase II detoxifying enzymes, suppress NLRP3 inflammasome assembly, and preserve cartilage integrity. Considering sulforaphane is already in clinical use as a nutraceutical, its safety profile [127] could facilitate the repurposing of this compound for CHIKV patients. Moreover, plant-derived alkaloids with both antiviral potency and radical-scavenging capacity, lycorine and berberine, display favourable pharmacokinetics in rodent models and are poised for first-in-human dose-escalation trials. The integration of redox biomarkers (MDA, protein carbonyls) in early-phase trials will be critical for establishing proof of mechanism and guiding combination regimens with standard analgesics. Early pharmacokinetic data indicate that oral dosing achieves synovial fluid concentrations sufficient for Nrf2 activation [128], paving the way for first‐in‐human trials that incorporate redox biomarkers (MDA, 4-hydroxynonenal) as surrogate endpoints [129].

Nrf2 activators and phenolic antioxidants reduce joint inflammation in animal models, yet uncertain human pharmacokinetics in synovial tissues may yield subtherapeutic exposures. Oral formulations often fail to achieve the tissue concentrations needed to sustain phase II enzyme induction over chronic arthralgia timelines [130]. Sustained Nrf2 activation carries theoretical risks, such as impaired immune surveillance and potential tumorigenesis, necessitating rigorous long-term safety studies [131]. The absence of standardized redox biomarker assays across clinical laboratories [132] further impedes the ability to monitor pharmacodynamics and correlate them with clinical outcomes. For example, F2-isoprostanes are often cited as the gold standard for lipid peroxidation studies [133]. While the mass spectrometry is frequently accurate, the variety of preparation protocols across laboratories leads to wildly different baseline values. This variance prevents clinicians from establishing a universal threshold to predict patient outcomes in cardiovascular disease or neurodegeneration. Another concern emphasized the gap between research-grade and clinical-grade biomarkers. For example, protein carbonyls used in different commercial ELISA-based kits provide non-comparable results. This is due to pharmacodynamics, which relies on measuring a change in a biomarker over time in response to a drug [134]. While a drug's redox-lowering effect might be significant in a Phase I trial at one site, it can be invisible in a Phase II trial at another site. On top of the given examples, the lack of specificity in Thiobarbituric Acid Reactive Substances (TBARS) assays [135], and the lack of reference ranges and quality control in measuring total antioxidant capacity (TAC) assays [136] also make it impossible to verify whether a patient's low result is a clinical risk or just an artefact.

Translating experimental redox interventions into clinical protocols requires a paradigm shift from static biomarker measurement toward standardized, dynamic redox profiling. Central to this shift is the adoption of *ex vivo* oxidative challenges to quantify a patient's functional redox buffer capacity, a metric that accounts for baseline physiological variability and defines a precise therapeutic window for antioxidant administration without compromising requisite early-phase antiviral ROS signalling [137]. The diagnostic reliability of these assessments can be further bolstered by the implementation of ratio-metric panels, such as the GSH/GSSG index [138], which minimize lab-to-lab variance and the confounding effects of plasma leakage, while point-of-care electrochemical paper-based analytical devices (ePADs) facilitate real-time pulse dosing based on TAC fluctuations [139]. Furthermore, the deployment of machine-learning-derived digital twins, which integrate standard haematological parameters with redox kinetics, allows for the predictive modelling of the critical transition from host-protective oxidative bursts to pathological tissue injury [140].

In neurodevelopmental injury, translation of redox therapies taps into an expanding repertoire of clinically advanced agents. Mitochondria-targeted peptides such as SS-31 (elamipretide), currently in phase II trials for other ischemic and degenerative conditions [141], directly scavenge mtROS, preserve membrane potential in neural progenitors, and reduce apoptosis in foetal brain explant models. PPAR-*γ* agonists, exemplified by rosiglitazone, not only correct lipid dysregulation but also upregulate endogenous antioxidants, offering a dual mechanism to protect the developing CNS. Coupling these agents with real‐time imaging of oxidative stress via PET tracers [142] or redox-sensitive MRI contrast agents [143] will enable patient stratification and endpoint assessment in clinical studies of pregnant women at risk of congenital Zika syndrome.

Mitochondria-targeted peptides and PPAR-*γ* agonists promise neuroprotection in foetal models, but stringent safety thresholds for maternal-foetal exposure present major regulatory barriers. Pre-clinical teratogenicity studies do not always predict human outcomes, making first-in-pregnancy trials ethically and logistically complex. Blood-brain barrier permeability varies dramatically among redox compounds [144], complicating dose selection for effective CNS penetration without systemic side effects. Experimental imaging techniques, such as redox-sensitive PET tracers, lack normative pregnancy data [145], making it difficult to stratify patients or define clear trial endpoints.

Despite the potent *in vitro* efficacy of redox-modulating agents discussed above, their clinical translation remained hindered by a critical lack of standardized biomarker assays and the intracellular bioavailability gap defined by the *pEC*_50_ = *pIC*_50_ + log *F*_*ic*_ framework [146]. Ultimately, successful therapeutic intervention requires a shift toward time-sensitive, tissue-specific strategies that prioritize intracellular accumulation (*F*_*ic*_) to arrest self-perpetuating cycles of oxidative damage and tissue-specific injury, ranging from vascular shock to neurodevelopmental defects and chronic arthritis, before irreversible clinical endpoints are reached.

The successful repositioning of agents such as Zidovudine (AZT) for HIV, Remdesivir and Paxlovid for COVID-19 [147,148], and the immunomodulator Baricitinib for severe viral inflammation [149] underscores a proven regulatory pathway for redirecting FDA-approved pharmacopoeias toward emerging infectious threats. However, the FDA has not yet approved a repurposed antiviral specifically for DENV, CHIKV, and ZIKV, leaving patient management largely restricted to supportive care. Current high-throughput screenings and pharmacometric evaluations are actively bridging this gap by identifying candidates that target both viral replication and the host's redox-sensitive pathways. Notable examples include Eltrombopag as a DENV NS2B-NS3 protease inhibitor [88], the synergistic use of Hepatitis C virus direct-acting antivirals, such as Simeprevir, for CHIKV nsP2 proteolysis [89], and the anti-asthmatic Montelukast for ZIKV [90]. By focusing on these existing compounds, including cardiovascular agents such as Candesartan [150] and the antidiabetic Empagliflozin [151], researchers aim to exploit their established safety profiles and known intracellular bioavailability to arrest mitochondrial remodelling and oxidative redox collapse that drive severe vascular leakage and neurological sequelae characteristic of these arboviral crises.

## 6. Conclusion

Advances in redox-based therapies underscore the feasibility of translating redox-modulating strategies into human trials. By targeting host oxidative pathways, these interventions not only blunt virus‐driven immunopathology, vascular leakage in dengue, chronic arthralgia in chikungunya, and neurodevelopmental injury in Zika but also enhance the efficacy of antiviral regimens through synergistic reduction of viral replication and collateral tissue damage. Nonetheless, translating redox‐modulating strategies into the clinical phases faces significant hurdles. The narrow therapeutic index of antioxidants risks impairing essential ROS-mediated antiviral defences, while inter-individual variation in baseline redox status complicates dose optimization. Pharmacokinetic challenges, such as achieving effective synovial or CNS concentrations without systemic toxicity, and ethical constraints, particularly in pregnant populations at risk for congenital Zika syndrome, further impede progress. The absence of validated, standardized assays for tissue-specific ROS measurement and redox biomarker endpoints undermines robust dose-response assessment in early-phase trials. To overcome these barriers, future research must prioritize the development and validation of redox-sensitive biomarkers, adopt advanced formulation platforms (PEGylation, liposomes, nanocarriers) for targeted tissue delivery, and design adaptive, biomarker-driven clinical trials. Leveraging compounds with established safety profiles and repurposing FDA-approved redox modulators can accelerate regulatory pathways. Integration of genomic and metabolomic profiling will refine patient stratification and inform personalized dosing regimens. Finally, cross-disciplinary consortia of virologists, pharmacologists, and clinical investigators are essential to harmonize assay standardization, establish clear translational milestones, and ultimately translate redox-based interventions into effective therapies that reduce the global morbidity of arboviral diseases.

## Supplementary Material

Supplementary material ISSM PRISMA Checklist_CKY.pdf
